# Valproic acid ameliorates coxsackievirus-B3-induced viral myocarditis by modulating Th17/Treg imbalance

**DOI:** 10.1186/s12985-016-0626-z

**Published:** 2016-10-10

**Authors:** Haibin Jin, Xiaoming Guo

**Affiliations:** Department of Geria Care, 254th Hospital of PLA, No 60 Huangwei Road, Tianjin, 300142 China

**Keywords:** Viral myocarditis, Valproic acid, Th17 cells, Treg cells

## Abstract

**Background:**

Viral myocarditis, which is often caused by coxsackievirus B3 (CVB3), is a serious clinical disorder characterized by excessive myocardial inflammation. Valproic acid (VPA) is described as a histone deacetylase inhibitor that has anti-inflammatory effects in several inflammatory diseases. However, the role and the detailed mechanism of VPA in viral myocarditis remain unclear.

**Methods:**

Experimental CVB3-induced myocarditis was induced in mice by intraperitoneally (i.p.) infected with CVB3. VPA was i.p. administered from day 0 to day 7. The survival, body weight loss, and myocarditis severity of mice were recorded. Th17 and Treg cells in spleen were analyzed by flow cytometry. Th17/Treg cell-related cytokine expressions were quantified by ELISA. The effect of VPA on Th17 and Treg cells differentiation was examined in vitro and in vivo.

**Results:**

Administration of VPA significantly attenuated the clinical severity of myocarditis, and the overall mortality from CVB3-induced myocarditis. The infiltration of Th17 and Treg cells, as well as the serum level of related cytokines (IL-17A and IL-10), were increased in CVB3 infected mice. In addition, VPA decreased the percentage of splenic Th17 cells while increased the percentage of Treg cells. Moreover, VPA downregulated the expression of IL-17A and upregulated IL-10 in serum and heart tissues of CVB3 infected mice. Additionally, VPA directly inhibited the differentiation of Th17 cells and promoted both the differentiation and suppressive function of Treg cells in vitro and in vivo.

**Conclusions:**

Our results suggest that VPA may thus be a promising strategy in the therapy of viral myocarditis.

## Background

Viral myocarditis, which is often caused by coxsackievirus B3 (CVB3)-triggered myocarditis, is one of the leading causes of severe heart failure, and even sudden death in young adults [1]. Viral myocarditis is an immune-mediated disease characterized by inflammatory infiltration of the myocardium and cardiac myocyte necrosis [2, 3]. It has been shown that several T helper (Th) subsets, such as Th17 and Treg cells, are involved in the pathogenesis of viral myocarditis [4–6]. Th17 cells are a new subset of CD4^+^ T cells that secrete the characteristic cytokine interleukin-17A (IL-17A). There is also evidence that Th17 cells contribute to myocarditis development and pathogenesis [4, 5, 7]. Treg cells characteristically express the transcription factor forkhead box P3 (Foxp3), secrete anti-inflammatory cytokines such as IL-10 and transforming growth factor-β (TGF-β), and act to maintain immune homeostasis and control immune-mediated tissue injury. Treg cells are also involved in inflammation and tissue destruction during viral myocarditis [4, 6]. More importantly, Th17 and Treg cells are subsets of the CD4^+^ T cell which are vital modulators of the innate and adaptive immune response in many immune diseases [8]. A Th17/Treg imbalance has been found in many diseases, including viral myocarditis [4, 9]. However, the expression and function of Th17 and Treg cells in viral myocarditis remain unclear.

Histone deacetylases (HDACs) are part of a vast family of enzymes involved in chromatin remodeling that play a key role in the differentiation and immune function of T cells [10, 11]. Inhibition of HDAC activity is initially recognized as a powerful anti-cancer therapeutic strategy and is recently found to be implicated in the regulation of the neurodegenerative diseases, and inflammatory diseases [12]. Valproic acid (VPA), a short-chain branched fatty acid, is a clinically approved HDAC inhibitor that is traditionally used in the treatment of epilepsy and has been shown to be a safe, effective treatment in humans [13]. Accumulated experimental and clinical data also show that VPA could be a potent anticancer drug, and has neuroprotective, axonal remodeling and anti-inflammatory effects. Lv et al. reported that VPA could inhibit experimental autoimmune encephalomyelitis in mouse model by inducing apoptosis in activated T cells and maintaining the immune homeostasis [14]. Administration of VPA could decrease disease incidence and severity in collagen induced mouse arthritis through effects on the production and function of Treg cells [15]. VPA could also suppress proinflammatory cytokine synthesis and reduce the severity of dextran sodium sulfate-induced colitis [16]. Thus, it is reasonable to hypothesize that HDAC inhibitor plays a protective role in viral myocarditis. One study showed that HDAC inhibitors could suppress CVB3 growth in vitro and CVB3-induced myocardial injury in vivo [17]. However, the role and the detailed mechanism of VPA in viral myocarditis remain unclear. In the current study, we observed that administration of VPA ameliorated CVB3-induced viral myocarditis by modulating Th17/Treg imbalance. Our findings suggest that VPA may be a promising therapeutic strategy for treating viral myocarditis.

## Methods

### Mice and virus

Six to eight week old male specific pathogen-free BALB/c mice were purchased from Vital River Laboratories (Beijing, China) and maintained at the Animal Center of the Nankai University (Tianjin, China). CVB3 (Nancy strain) was propagated in HeLa cells. The viral titer was determined using a 50 % tissue culture infectious dose (TCID50) assay on HeLa cell monolayers and calculated by the Reed-Muench method. This study was carried out in strict accordance with the recommendations in the Guide for the Care and Use of Laboratory Animals of the National Institutes of Health. The experimental protocol was approved by the Committee on the Ethics of Animal Experiments of the Nankai University (Permit Number: 15–1567).

### CVB3 infection and drug administration

BALB/c mice were infected with an intraperitoneal (i.p.) injection of 0.1 mL of phosphate-buffered saline (PBS) containing 10^3^ TCID_50_ of the virus on day 0. Mice administered i.p. with PBS were taken as mock control. Mice were administered VPA (250 mg/kg; Selleck) or vehicle solution (PBS) by i.p. injection daily from day 0 to day 7.

### Histopathology and myocarditis scoring

Mice hearts were fixed in 10 % formalin, paraffin embedded, and stained with hematoxylin and eosin (H&E). Severity scores of myocarditis were graded by two independent pathologists in a double-blinded manner based on the following semi-quantitative scale: 0 = no inflammation; 1 = one to five distinct mononuclear inflammatory foci, with the involvement of 5 % or less of the cross-sectional area; 2 = more than five distinct mononuclear inflammatory foci, or the involvement of over 5 % but not over 20 % of the cross-sectional area; 3 = diffuse mononuclear inflammation involving over 20 % of the area, without necrosis; and 4 = diffuse inflammation with necrosis.

### Flow cytometry

Single-cell suspensions were obtained from spleen of mice on indicated days postinfection. For intracellular cytokine staining, cells were stimulated for 5–6 h with PMA (50 ng/mL) and ionomycin (500 ng/mL) in the presence of brefeldin A (10 μg/ml). Cells were harvested, washed, and stained with surface molecule antibodies in the presence of FcR-Block (BD Bioscience). After the wash, cells were then fixed using CytoFix/CytoPerm buffer (BD Bioscience) and stained with antibodies against intracellular cytokines or isotype control on ice for 30 min. The following Abs were used for staining: FITC-conjugated anti-mouse CD3, PE-conjugated anti-mouse IL-17A, PE-Cy5.5-conjugated anti-mouse IFN-γ, and APC-conjugated anti-mouse CD4 (BD Bioscience). Treg cell staining was performed using a Treg staining kit according to the manufacturer’s instructions (eBioscience). Data were acquired on a FACS Calibur (BD Bioscience) and analyzed using Flowjo software (TreeStar).

### Real-time PCR

Total RNA from heart tissues was extracted with TRIzol Rreagent (Invitrogen), and reverse transcripted into cDNA. Transcription levels of *Il17a* and *Il10* genes were analyzed by real-time quantitative PCR using an ABI 7500 (Applied Biosystems) and SYBR green system (TaKaRa) by following the manufacturer’s protocol. The primers used were: *Il-17a*, forward: 5’-CTCCAGAAGGCCCTCAGACTAC-3’ and reverse: 5’-AGCTTTCCCTCCGCATTGACACAG-3’; *Il10*, forward: 5’- GACAACATACTGCTAACCGACTC-3’ and reverse: 5’- GACAACATACTGCTAACCGACTC-3’; CVB3, forward: 5’- GCACACACCCTCAAACCAGA -3’ and reverse: 5’- ATGAAACACGGACACCCAAAG -3’; GAPDH, forward: CTCTGGAAAGCTGTGGCGTGATG-3’ and reverse: 5’- ATGCCAGTGAGCTTCCCGTTCAG-3’. The relative gene expressions were normalized to the level of GAPDH and quantified by the 2^-△△CT^ method. All reactions were performed in at least duplicate for each sample.

### Western blotting

About 20 μg of total protein form heart tissues was extracted from CVB3 infected mice, and separated by 10 % SDS-PAGE, transferred onto polyvinylidene fluoride membranes. Membranes were blocked with nonfat dry milk solution (5 % in Tris-buffered saline) containing 0.1 % Tween 20 for 1 h, and then incubated with mouse anti-Enterovirus VP1 antibody (clone 5-D8/1, Dako, Hamburg, Germany) overnight at 4 °C. After being washed with PBS containing 0.1 % Tween 20, the membranes were incubated with Horseradish peroxidase (HRP)-conjugated goat anti-mouse antibody for 1 h at room temperature. The bands were visualized by enhanced chemiluminescence (Beyotime, Shanghai, China) and detected by an Alpha Imager (Alpha Innotech, San Leandro, CA, USA).

### ELISA

Levels of IL-17A and IL-10 in serum or cell culture supernatants were determined by ELISA kits (eBioscience) according to the manufacturer’s instructions. All samples were measured in triplicate.

### In vitro T cells differentiation

Naive CD4^+^T cells from normal BALB/c mice were purified using CD4^+^ naive T cell isolation kit (STEMCELL Technologies) according to the manufacturer’s instruction. Purified cells were activated by plate-coating anti-CD3 (10 μg/ml; BD Pharmingen) plus anti-CD28 (2 μg/ml; BD Pharmingen) for 5 days under the following polarizing conditions: TGF-β (3 ng/ml, Peprotech), IL-6 (30 ng/ml; eBioscience), IL-23 (20 ng/ml; R&D), anti-IFN-γ (10 μg/ml, BD Pharmingen), anti-IL-4 (10 μg/ml, BD Pharmingen) for Th17 polarization, and TGF-β1 (5 ng/ml, Peprotech), anti-IFN-γ (10 μg/ml, BD Pharmingen), anti-IL-4 (10 μg/ml, BD Pharmingen) for Treg polarization. VPA or vehicle solution was added on day 0.

### In vitro suppression assay

CD4^+^CD25^+^ T cells were isolated from the spleen of CVB3 infected mice treated with VPA or vehicle on day 6 using Mouse CD4^+^CD25^+^ Regulatory T Cell Isolation Kit (STEMCELL Technologies). CD4^+^CD25^−^ T cells were used as T effector (Teff) cells and labeled with 1 μM CFSE (Life technologies). Purified Treg cells (5 × 10^4^ cells) were cultured with Teff cells at 1:2 ratios in 96-well round bottom plates coating with anti-CD3 (10 μg/ml; BD Pharmingen) plus anti-CD28 (2 μg/ml; BD Pharmingen). Ninety-six hour later, the suppression was assayed by FACS analysis for dilution of CFSE in gated Teff cells. Data were acquired on a FACS Calibur (BD Bioscience) and analyzed using Flowjo software (TreeStar).

### Statistical analysis

All Data were presented as means ± SEM. Differences between experimental groups were analyzed using an unpaired Student’s *t*-test. Survival was estimated by the Kaplan-Meier method and compared by the log-rank test. All data were analyzed with GraphPad Prism version 5.0 (GraphPad Software Inc.). *P* < 0.05 was considered significant difference.

## Results

### VPA treatment attenuates CVB3-induced myocarditis in mice

**Fig. 1:**
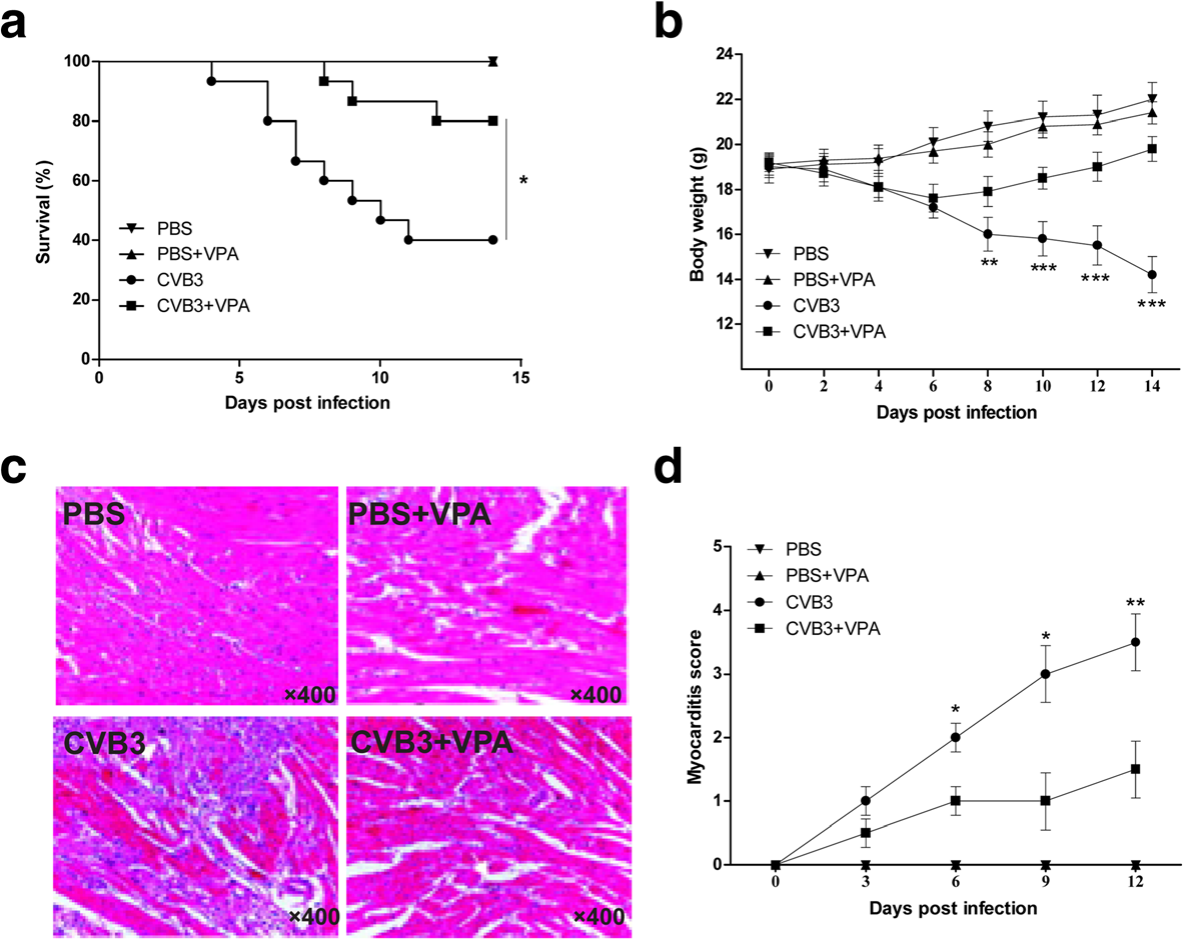
Administration of VPA attenuates CVB3-induced myocarditis in mice. Male BALB/c mice (*n* = 20 per group) were infected with PBS or CVB3 on day 0, and then treated with either VPA (i.p., 250 mg/kg) or vehicle (PBS) daily from day 0 to day 7 postinfection. The survival rate (**a**) and body weight change (**b**) were monitored daily until day 14. Paraffin sections of heart tissues were prepared on day 0, 3, 6, 9, 12 postinfection and cardiac injury was revealed by H&E. Representative H&E on day 6 postinfection was shown (**c**). The myocarditis pathological score was determined by analysis of the H&E-stained sections (**d**). Results are presented as the mean ± SEM of three independent experiments. ^*^ *P* < 0.05, ^**^ *P* < 0.01, ^***^ *P* < 0.001, compared with CVB3 group

To investigate the effect of VPA on CVB3-induced viral myocarditis, mice were first infected with CVB3 or mock and then treated daily with either VPA (i.p., 250 mg/kg) or vehicle from day 0 to day 7 after infection. As expected, mice receiving PBS alone developed no sign of viral myocarditis, whereas the signs of viral myocarditis were apparent in CVB3-infected mice, including weakness, irritability, lethargy and weight loss, with 40 % of mice died on day 14 postinfection (Fig. 1a). In contrast, the administration of VPA significantly ameliorated CVB3-induced myocarditis in CVB3-infected mice, with ~ 80 % of them surviving without severe viral myocarditis (*P* = 0.02, Fig. 1a). This was accompanied by less body weight loss (Fig. 1b). The pathological myocarditis score also was lower in VPA treated CVB3-infected mice (Fig. 1c and d). However, administration of VPA to mock-infected mice caused no mortality, bodyweight loss, and cardiac pathological analysis showed no evidence of viral myocarditis (Fig. 1a–d). Taken together, VPA treatment attenuates CVB3-induced myocarditis in mice without s systemic toxicity.

### VPA administration regulates the proportion of Th17 and Treg cells

**Fig. 2:**
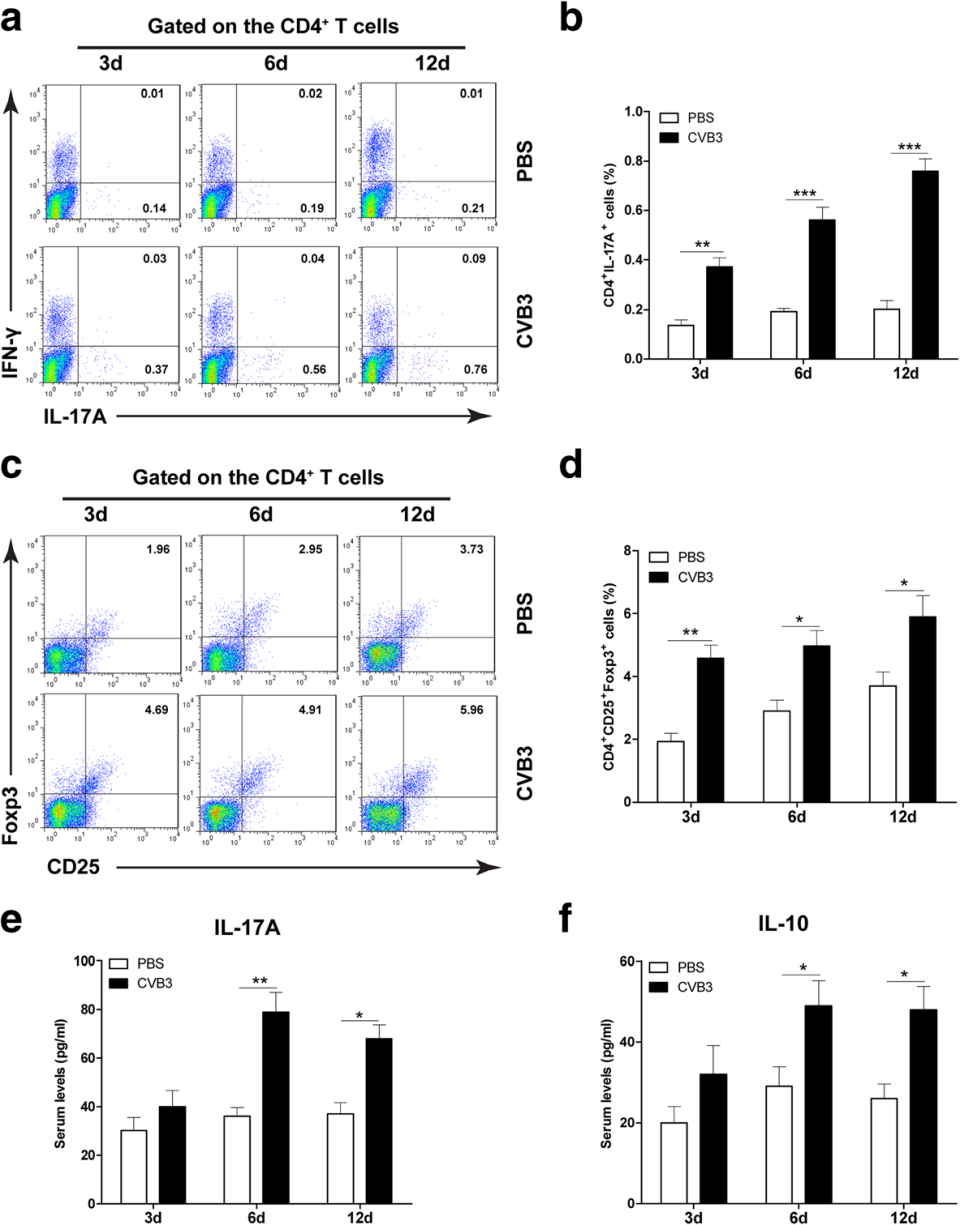
Th17 and Treg cells are involved in CVB3-induced myocarditis. Male BALB/c mice (*n* = 20 per group) were infected with PBS or CVB3 on day 0. The frequencies of CD4^+^IL-17^+^ Th17 cells or CD4^+^CD25^+^Foxp3^+^ Treg cells in mice from spleen on indicated days postinfection were analyzed by Flow cytometry (**a** and **c**, *left panels*). Bar graphs showed the representative percentages of Th17 cells or Treg cells (**b** and **d**, *right panel*). The serum levels of characteristic cytokines IL-17A (**e**) and IL-10 (**f**) in CVB3 infection mice were determined by ELISA. Results are presented as the mean ± SEM of three independent experiments. ^*^ *P* < 0.05, ^**^ *P* < 0.01, ^***^ *P* < 0.001, compared with PBS group

Increasing evidence has demonstrated that a balance between proinflammatory Th17 and anti-inflammatory Treg cells is essential to maintain immune tolerance and prevent the onset of several inflammatory and autoimmune diseases [8]. Therefore, to understand the mechanism by which VPA treatment reduced CVB3-inducedmyocarditis, we first monitored the kinetics of T cell proportion during CVB3 infection. As shown in Fig. 2a and b, in the CVB3-infected mice, CD4^+^IL-17^+^ Th17 cells derived from spleen significantly increased since the early stage after CVB3 infection compared with the mock-infected mice. In addition, the percentages of CD4^+^CD25^+^Foxp3^+^Treg cells in CVB3 infected mice were also higher than those in the mock control mice (Fig. 2c and d). Moreover, the characteristic cytokines produced by Th17 (IL-17A) and Treg (IL-10) cells were elevated in serum from CVB3 infected mice (Fig. 2e and f). These data suggest that Th17 and Treg cells are involved in CVB3-induced myocarditis.

**Fig. 3:**
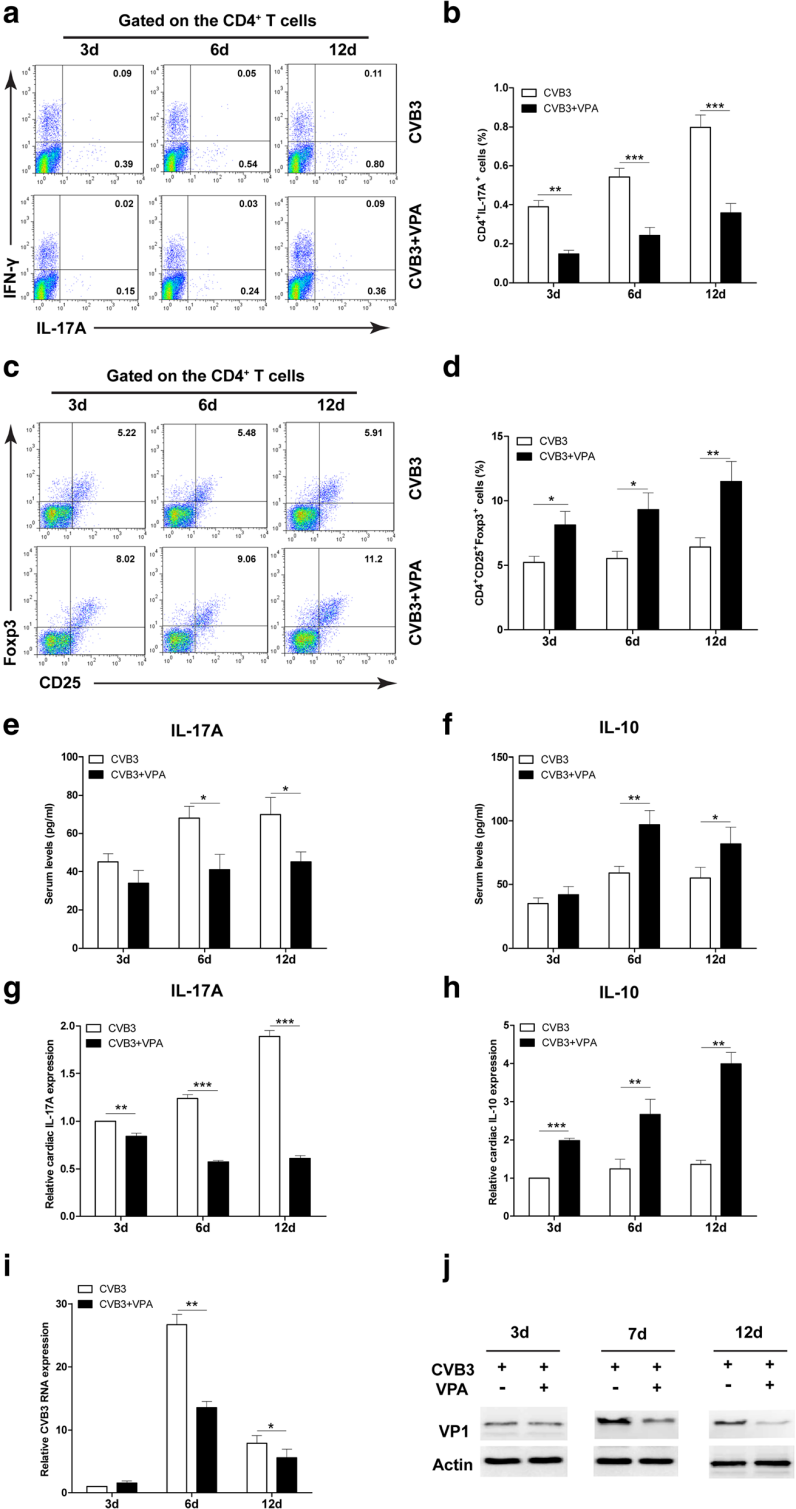
Administration of VPA regulates the proportion of Th17 and Treg cells. Male BALB/c mice (*n* = 20 per group) were infected with CVB3 on day 0, and then treated with either VPA (i.p., 250 mg/kg) or vehicle (PBS) daily from day 0 to day 7 postinfection. The frequencies of Th17 cells or Treg cells in mice from spleen on indicated days postinfection were analyzed by Flow cytometry (**a** and **c**, *left panels*). Bar graphs showed the representative percentages of Th17 cells or Treg cells (**b** and **d**, *right panel*). The serum levels of characteristic cytokines IL-17A (**e**) and IL-10 (**f**) in CVB3 infection mice were determined by ELISA. mRNA expression of IL-17A (**g**) and IL-10 (**h**) in cardiac tissues were detected by qRT-PCR on indicated days postinfection. **i** The CVB3 replication in cardiac tissues were detected by qRT-PCR on indicated days postinfection. **j** The expression of viral capsid protein (VP1) in cardiac tissues was detected by western blot on indicated days postinfection. Results are presented as the mean ± SEM of three independent experiments. ^*^ *P* < 0.05, ^**^ *P* < 0.01, ^***^ *P* < 0.001, compared with CVB3 group

We then treated CVB3 infected mice with VPA or vehicle and analyzed the proportion of Th17 and Treg cells from spleen on the indicated days after CVB3 infection. Flow cytometry analysis showed that, compared with vehicle control, VPA treatment markedly reduced the frequencies of splenic Th17 cells (Fig. 3a and b), whereas increased the frequencies of Treg cells in the spleen on day 3, 6 and 12 (Fig. 3c and d). We also detected substantially lower serum levels of Th17-related cytokines (IL-17A) and higher serum levels of Treg-related cytokines (IL-10) in VPA treated mice during CVB3 infection (Fig. 3e and f). Additionally, the mRNA expressions of cardiac IL-17A in VPA mice were markedly reduced compared to the vehicle control mice (Fig. 3g); whereas the mRNA levels of cardiac IL-10 in the VPA mice were apparently elevated compared to the vehicle control mice (Fig. 3h). We also examined the effect of VPA on CVB3 viral replication in vivo, the results showed that VPA significantly reduced virus replication (Fig. 3i) and viral protein VP1 expression (Fig. 3j) in heart tissues of VPA treated mice during CVB3 infection. Taken together, our data suggest that the decrease in Th17 cells and increase in Treg cells, as well as decreased CVB3 viral replication may be the major causes of VPA-mediated viral myocarditis inhibition.

### VPA influences Th17 and Treg cells differentiation in vitro and in vivo

**Fig. 4:**
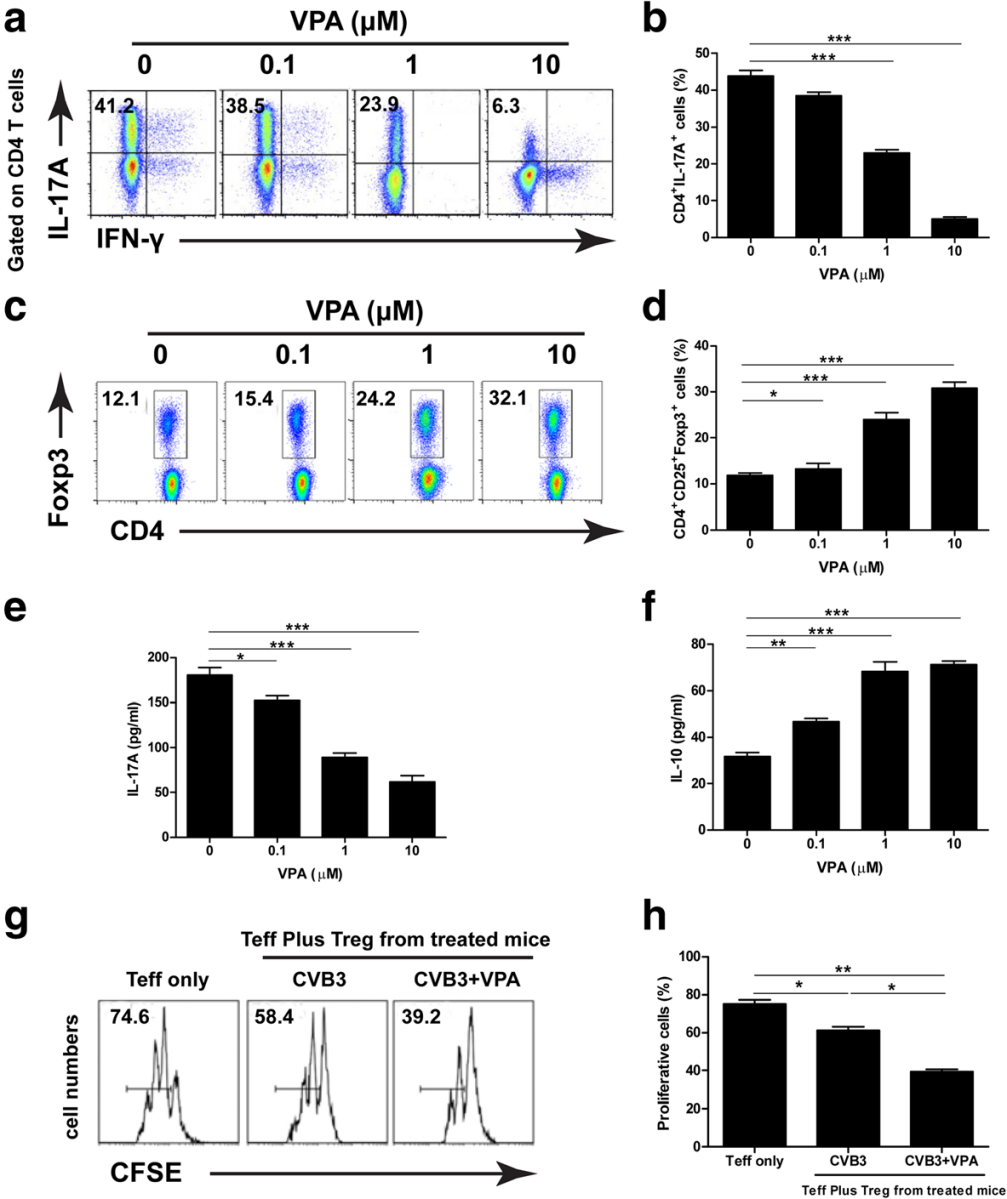
VPA influences Th17 and Treg cells differentiation in vitro and in vivo*.* Purified naive CD4^+^ T cells from normal BALB/c mice were cultured with different concentrations of VPA under Th17 or Treg cells-polarizing conditions. The differentiations of Th17 cells or Treg cells were analyzed by Flow cytometry (**a** and **c**, *left panels*). Bar graph shows the representative percentage of in vitro-differentiated Th17 cells or Treg cells (**b** and **d**, *right panel*). Spleen CD4^+^ T cells isolated from mice 6d after CVB3 infection were treated with PBS or VPA for 24 h ex vivo, the supernatants were collected and ELISA was performed to determine the level of IL-17A (**e**) and IL-10 (**f**). CD4^+^CD25^+^ T cells isolated from CVB3 infected mice treated with VPA or vehicle were isolated on day 6 and co-cultured with CFSE-labeled CD4^+^CD25^−^T effector (Teff) cells from naïve mice on anti-CD3/CD28 mAb coated plates. The suppression was assayed by Flow cytometry for dilution of CFSE in gated Teff cells (**g** and **h**). Results are presented as the mean ± SEM of three independent experiments. ^*^ *P* < 0.05, ^**^ *P* < 0.01, ^***^ *P* < 0.001

VPA is known to directly inhibit class I and II HDAC activity and cause a hyperacetylation of histones. Since histone acetylation pattern remodeling has been found to associate with T cell differentiation and cytokines expression [18], we proposed that VPA may influence Th17 and Treg cells differentiation directly. Firstly, purified naive CD4^+^ T cells from normal BALB/c mice were cultured under Th17 or Treg cells-polarizing conditions with or without VPA. As shown in Fig. 4a–d, VPA inhibited the in vitro differentiation of Th17 cells, whereas promoted Treg cells differentiation in a dose depend manner.

We next examined whether VPA directly influences the immune function of in vivo-differentiated Th17 and Treg cells. Spleen CD4^+^ T cells were isolated from mice 6d after CVB3 infection and cultured with or without VPA for 24 h to determine cytokine production. Data showed that VPA reduced IL-17A and induced IL-10 production in a dose-dependent manner (Fig. 4e and f). Furthermore, we assessed the effect of VPA on immune function of Treg cells. CD4^+^ CD25^+^ T cells from CVB3 infected mice treated with VPA or vehicle were isolated and co-cultured with CFSE-labeled CD4^+^CD25^−^T effector cells from naïve mice on anti-CD3/CD28 mAb coated plates. As shown in Fig. 4g and h, the percentage of proliferative Teff cells was reduced from 74.6 % (in the absence of Treg) to 58.4 % by the addition of Treg from vehicle-treated mice. However, in the presence of VPA-treated Treg, the population of proliferating Teff cells was further reduced to 39.2 %, indicating an enhanced Treg suppressive function in CVB3 infected mice following VPA treatment. Altogether, these data suggest that VPA directly inhibits the differentiation of Th17 cells and promotes both the differentiation and suppressive function of Treg cells in vitro and in vivo.

## Discussion

In this study, we provided the first evidence that administration of VPA protected against CVB3 induced viral myocarditis. We also observed an imbalance of Th17/Treg cells during the CVB3 infection. The infiltration of Th17 and Treg cells, as well as the serum level of related cytokines, also was increased in CVB3 infected mice. In addition, the protective effects of VPA against viral myocarditis were closely associated with Th17/Treg imbalance modulation and decreased CVB3 viral replication during the pathogenesis of viral myocarditis. VPA directly inhibited the differentiation of Th17 cells and promoted both the differentiation and suppressive function of Treg cells in vitro and in vivo, thus possibly providing a protective effect against viral myocarditis. Therefore, our results suggest that VPA may thus be a promising strategy to prevent or treat viral myocarditis.

Many studies reported that Th17/Treg functional imbalance exists during acute CVB3-induced myocarditis, Treg and proinflammatory Th17 cells have been suggested to play either suppressor or effector roles, respectively. Xie et al. showed that acute myocarditis exhibited apparent increases in Th17 cells and Th17-related cytokines (IL-17A, IL-21). Meanwhile, marked increases in Treg cells and Treg-related cytokines (TGF-β, IL-10 and IL-6) also were observed in myocarditis mice [4]. They further demonstrated that blockade of IL-17A protected against CVB3-induced myocarditis by increasing COX-2/PGE2 production in the heart [7]. On the other hand, Cao et al. found that adoptive transfer of Treg cells protected against CVB3-induced cardiac fibrosis [6]. Consistent with these studies, in the present study, we also found an increased percentage of Th17 cells and Treg cells in CVB3 infected mice from early stage of infection, suggesting that Th17 and Treg cells are involved in CVB3-induced myocarditis. Thus, personalized medicine that alters the balance between Treg and Th17 cells may ameliorate viral pathology during CVB3 infections.

VPA is known to interfere with multiple regulatory mechanisms, including class I and II HDACs, Akt, ERK, and NF-κB pathway [19, 20]. Recently, VPA has been found to have anti-inflammatory effects that are probably associated with its HDAC inhibitory activity [21]. Histone acetylation remodeling has been found to associate with T cell differentiation and expression of a variety of cytokines [18]. HDAC inhibitors effectively reduced the expression of pro-inflammatory cytokines, including IL-1β, IFN-γ, TNF-α, and IL-6 [11]. Therefore, VPA may function through the HDAC inhibitory activity to attenuate inflammation in viral myocarditis. In addition, Shim et al. reported that Histone deacetylase inhibitors Trichostatin A could suppress CVB3 replication in Hela cells in vitro, and subsequently reduces CVB3 titers in the heart and protects mice from myocarditis [17]. In our study, we also observed significantly decreased CVB3 replication in heart tissues in vivo, suggesting that decreased viral replication is also necessary for VPA-mediated amelioration of viral myocarditis. More importantly, administration of VPA ameliorated viral myocarditis accompanied by a marked reduction in the frequency of Th17 cells and relative cytokines, VPA can directly inhibit the in vitro and in vivo differentiation of Th17 cells. In addition, Foxp3 has an essential role in the development and function of natural and induced Treg and as such represents a key target to modulate Treg functions. Acetylation of Foxp3 is linked to stability of Foxp3 that can be regulated by HAT (i.e. CBP/p300 and TIP60) and HDAC (i.e. HDAC7, HDAC9 and SIRT1) [22, 23]. For example, in vivo treatment with VPA increases the number and function of Treg cells and reduces disease severity in the collagen induced mouse arthritis [15]. In humans, HDAC inhibition (vorinostat) regulates inflammation and enhances Tregs after allogeneic hematopoietic cell transplantation through increased STAT 3 acetylation and induced indoleamine-2,3-dioxygenase [24]. In our study, VPA treatment increased the percentage of Treg cells in CVB3 infected mice, and promoted both the differentiation and suppressive function of Treg cells in vitro and in vivo. Thus, modulation of Th17/Treg cell differentiation and immune function may represent a mechanism by which VPA controls viral myocarditis.

## Conclusions

In summary, we have studied the effect of VPA in CVB3 induced myocarditis, an animal model of human viral myocarditis. Our data demonstrate that VPA treatment greatly reduced the severity and duration of viral myocarditis and attenuated inflammation in the heart. Furthermore, VPA treatment in viral myocarditis suppressed the polarization of Th17 cells but induced the Treg cells. Therefore, our investigation showed that VPA could effectively ameliorate viral myocarditis through modulating Th17/Treg imbalance, suggesting that VPA may be an option in the therapy of viral myocarditis.
